# Hepatitis E Virus Infection in Patients with Systemic and Cutaneous Lupus Erythematosus

**DOI:** 10.3390/ijms252011162

**Published:** 2024-10-17

**Authors:** Fulvia Ceccarelli, Maria Dorrucci, Carmelo Pirone, Elida Mataj, Cristina Garufi, Francesca Farchi, Roberto Bruni, Umbertina Villano, Elisabetta Madonna, Giancarlo Iaiani, Massimo Ciccozzi, Anna Rita Ciccaglione, Fabrizio Conti, Alessandra Lo Presti

**Affiliations:** 1 Lupus Clinic, Rheumatology, Dipartimento di Scienze Cliniche, Internistiche, Anestesiologiche e Cardiovascolari, Sapienza Università di Roma, 00185 Rome, Italy; fulviaceccarelli@gmail.com (F.C.); pirone.carmelo@gmail.com (C.P.); cristinagarufi@gmail.com (C.G.); fabrizio.conti@uniroma1.it (F.C.); 2 Department of Infectious Diseases, Istituto Superiore di Sanità, 00161 Rome, Italy; maria.dorrucci@iss.it (M.D.); francesca.farchi@iss.it (F.F.); roberto.bruni@iss.it (R.B.); umbertina.villano@iss.it (U.V.); elisabetta.madonna@iss.it (E.M.); annarita.ciccaglione@iss.it (A.R.C.); 3 Institute of Public Health (ISHP), 1001 Tirana, Albania; elidamata@yahoo.com; 4 Department of Tropical and Infectious Diseases, Aou Policlinico Umberto I, 00161 Rome, Italy; giancarloiaiani@gmail.com; 5 Unit of Medical Statistics and Molecular Epidemiology, University Campus Bio-Medico of Rome, 00128 Rome, Italy; m.ciccozzi@unicampus.it

**Keywords:** hepatitis E virus, systemic lupus erythematosus, cutaneous lupus erythematosus, serological and epidemiological evaluation

## Abstract

Systemic lupus erythematosus (SLE) is a chronic autoimmune disease characterized by a multifactorial etiology in which genetic and environmental factors interplay. An exclusively cutaneous condition has been described and defined as cutaneous lupus erythematosus (CLE). In Italy, a nationwide blood donor survey found an overall HEV prevalence of 8.7%, with an interregional variation from 2.2% to 22.8%. In this study, we aimed to estimate HEV seroprevalence in a cohort of patients affected by SLE and CLE attending the Lupus Clinic, Sapienza University of Rome. Serum samples were tested for anti-HEV immunoglobulin Ig G and M antibodies using commercial enzyme-linked immunosorbent assay (ELISA) kits. Statistical analysis was performed. In total, 138 patients were enrolled, 92 (67%) affected by SLE and 46 by CLE. The prevalence of HEV infection was 23.9% in the CLE group and 7.6% in the SLE group. The anti-HEV+ prevalence was significantly more frequent in CLE. Some mechanisms may be linked to increased susceptibility to HEV such as a molecular mimicry associated with the CLE condition or with the skin compartment/skin self-antigens, as well as the involvement of the genetic background. Regarding the possible risk factors, no association was found, although, of note, the odds of HEV+ relative to contact with animals and to eating raw seafood were strongly higher than the unit in the CLE group.

## 1. Introduction

Systemic lupus erythematosus (SLE) is a chronic autoimmune disease characterized by a multifactorial etiology in which genetic and environmental factors (such as infections, ultraviolet radiation, air pollution, smoke, other respiratory exposures, diet, microbiome and many others) interplay to determine the disease development [1,2]. Several environmental factors have been implicated in determining SLE. In particular, the role of viral infections has been extensively evaluated, observing an epidemiological association and suggesting possible pathogenic mechanisms [3]. The chronic inflammation observed in SLE leads to autoantibody production, complement activation and immune-complex deposition, finally resulting in tissue damage [1].

In addition to the systemic disease, an exclusively cutaneous condition has been widely described: so-called cutaneous lupus erythematosus (CLE) [4,5]. Hepatitis E virus (HEV) is a small, non-enveloped, single-stranded ribonucleic acid (RNA) virus belonging to the Hepeviridae family, Orthohepevirus genus and Orthohepevirus A species [6,7,8,9]. There are different HEV genotypes. HEV-1 and -2 are prevalent in low- and middle-income areas where transmission is mainly fecal–oral. HEV-3 is spread worldwide, whereas HEV-4 is prevalent in Asia but also present in Europe. Usually, HEV-3 and -4 are transmitted by food through the ingestion of raw or undercooked meat and organs (especially liver and offal) of infected host animals (mostly pig, wild boar, deer and rabbit), or by direct contact with infected animals, affecting workers in pig farms or in slaughterhouses and hunters. Although there is no definitive evidence, food-borne transmission might also occur by consuming fecally contaminated vegetables, fruits, mollusks and drinking water [10].

A heterogeneous prevalence of anti-HEV IgG (from <5% to >50%) has been highlighted in the general population and among blood donors in different countries [9,11,12]. In Italy, a nationwide blood donor survey found an overall prevalence of 8.7%, with an interregional variation from 2.2% to 22.8% [13]. More recent data [10] identified a considerable variation in anti-HEV sero-prevalence among blood donors in the different Italian regions with higher values in Abruzzo (27%) and lower in Calabria (1.3%) [10].

The prevalence of this specific infection in patients affected by autoimmune diseases has been scarcely investigated, and, to our knowledge, no data are available so far concerning systemic and cutaneous lupus erythematosus cohorts.

Indeed, in the present study, we aimed to estimate HEV seroprevalence in a cohort of patients affected by SLE and CLE attending the Lupus Clinic of the Rheumatology Unit, Sapienza University of Rome. Moreover, by using a case–case study design, we aimed to assess possible associations between HEV infection and epidemiological/clinical features of both conditions.

## 2. Results

### 2.1. Characteristics of the Study Population

A total of 138 patients were enrolled, 92 (67%) affected by SLE and the remaining 46 (33%) by CLE. As expected, females were more represented in the SLE group than in the CLE group (91.3% vs. 71.7%, *p*-value: 0.003). The median age of the study population was 50 years (IQR range: 40–58 and age range: 18–74), with a median age of CLE patients slightly higher than those with SLE (52 vs. 47, *p*-value: 0.038) (Table S1). The majority of patients were Italians, who were more represented in the CLE group (100% vs. 88%, *p*-value: 0.016) (Table S1). All the foreign patients (N = 11) were affected by SLE; specifically, 54.5% were Caucasian (Hungary, Bulgaria, Romania and Albania), 27.3% Hispanics/South Americans (Peru and Brazil) and 18.2% Asiatic (China and Sri Lanka). All foreign patients had lived in Italy for at least 12 years.

In the SLE cohort, anti-double-stranded DNA (dsDNA) antibodies were detected in 62.0% of patients and anti-Ro/Sjögren-syndrome-related antigen A (anti-SSA) in 47.8%. Regarding CLE, antinuclear antibodies (ANAs) were found in 54.3% patients and anti-SSA in 19.6%; as expected, anti-phospholipid antibodies were more frequently detected in SLE than in CLE patients (Table S1).

Unsurprisingly, SLE patients experienced more treatment lines than CLE patients (Table S1). In particular, the prevalence of subjects treated by glucocorticoids was significantly higher in the SLE group (89.1%) than in the CLE group (23.9%, *p* < 0.001). Furthermore, the proportion of patients treated by hydroxychloroquine was similar in the two groups (SLE 95.6% vs. CLE 91.3%, *p* = NS, Table S1). In regard to work activities and education, no statistically significant differences were found between the two groups.

### 2.2. Hepatitis E Seroprevalence in SLE and CLE Patients

The prevalence of HEV infection (i.e., patients testing anti-HEV+ IgG) was 23.9% in the CLE group (11 individuals) and 7.6% (7 individuals) in the SLE group. Only 9.1% (1/11) of the foreign patients (all belonging to the SLE group) resulted in anti-HEV+ IgG.

Univariable and multivariable analyses for CLE vs. SLE patients are shown in Table 1. The univariable analysis indicated that the anti-HEV+ prevalence was significantly more frequent in CLE (the unadjusted OR of CLE vs. SLE = 3.82, 95% CI: 1.37–10.65; *p* = 0.010), as well as in the male gender (the unadjusted OR of CLE vs. SLE = 4.14, 95% CI: 1.57–10.89; *p* = 0.004). When adjusting for age and gender, the OR of CLE vs. SLE for anti-HEV+ was still greater than the unit, although not statistically significant (the adjusted OR of CLE vs. SLE = 2.88, 95% CI: 0.96–8.68; *p* = 0.060).

**Figure 1:**
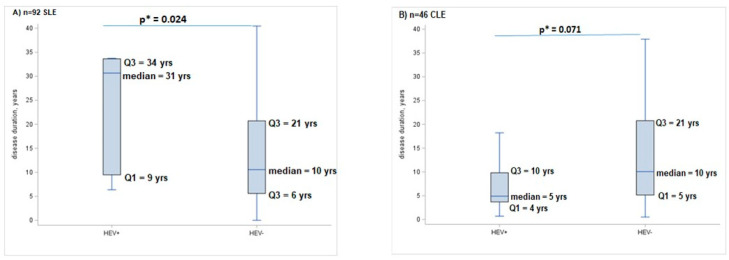
Box-plots showing the first (Q1), the second (median) and the third (Q3) quartile of the disease duration in years (yrs) according to HEV+ or HEV− in SLE (part (**A**); *p*-value = 0.024) and CLE group (part (**B**); *p*-value = 0.071); the difference between the two groups was assessed by * non-parametric Mann–Whitney U test.

In SLE subjects, we found a significantly longer disease duration in the anti-HEV+ group compared to anti-HEV− (median values 31 vs. 10 years, *p* = 0.024) (Figure 1A). On the contrary, in the CLE group, the disease duration was higher in anti-HEV− patients (10 vs. 5 years, *p* value = 0.071) (Figure 1B) but did not reach statistical significance.

Moving to clinical manifestations, anti-HEV+ SLE patients showed a lower prevalence of cytopenia compared to anti-HEV− (14.3% vs. 56.5%, *p* = 0.04).

Moreover, hypocomplementemia was more frequent in anti-HEV− patients compared to those with HEV+ (65.9% vs. 28.6%, *p* = 0.096) but without statistical difference; conversely, in the CLE group, hypocomplementemia was more frequent in the anti-HEV+ group, although it was not significant (18.2% vs. 8.6% *p* = 0.58).

### 2.3. Factors Associated with HEV Infection among SLE and CLE Patients

No association was reported with HEV+ considering one of the possible risk factors shown in Table 2, determined separately in the two groups (SLE and CLE). Of note, the OR of HEV+ relative to contact with animals and to eating raw seafood was strongly higher than the unit in the CLE group (Table 2).

## 3. Discussion

The present study represents the first attempt to investigate the seroprevalence of HEV infection in an Italian Lupus cohort, by using a case–case study design, and to assess possible associations between HEV infection and the epidemiological/clinical features of both conditions.

Previous studies, conducted in other countries evaluated the prevalence of HEV in autoimmune diseases, though it included very few Lupus patients [14,15,16].

However, the correlation between HEV infection and autoimmune diseases has been recently suggested. In particular, HEV infection has been linked to autoimmune hepatitis, Henoch–Schönlein purpura and thyroid diseases, including autoimmune thyroiditis, subacute thyroiditis and hyperthyroidism [17,18,19,20,21,22]. In the present study, we found a higher prevalence of HEV infection (i.e., patients testing anti-HEV+ IgG) in the CLE group compared to the SLE cohort. Indeed, in our study, we found an association between anti-HEV+ positivity and CLE, as well as male gender. Even without confirmation after adjusting for age and gender, our results can still provide a preliminary indication of a possible association between exposure to HEV and CLE, even if a specific HEV− control group was not included.

CLE is generally characterized by isolated skin findings. The pathogenic mechanism determining CLE development is not fully understood, but it is likely multifactorial, where a combination of genetic and environmental features (such as infections, ultraviolet radiation, air pollution, smoke, other respiratory exposures, diet, microbiome and many others) can act [23,24]. In order to explain our data, it is important to consider the differences between the CLE and SLE pathological conditions and the specific aspects related to the hepatitis E infection prevalence.

Some authors have tried to explore the risk factors that define CLE and to characterize the relationship between the pathogenesis of CLE and that of SLE, i.e., the level of IFN gene expression correlated with cutaneous disease activity (representing a possible biomarker for CLE) [24] and the TNFα expression in the skin and sera of CLE patients [25]. Some of these factors may be linked to increased susceptibility to HEV infection, as well as the involvement of the genetic background.

Moreover, HEV has been previously associated with another autoimmune disorder, the Guillain–Barre syndrome, with two possible proposed mechanisms: the first one is through direct viral damage due to HEV replication, and the second one is through molecular mimicry between infectious agents and peripheral nerve self-antigens, which may result in nerve injury [26]. It is possible to speculate a similar mechanism to explain our data, i.e., a mimicry associated with the CLE condition or to the skin compartment/skin self-antigens. Another aspect to be considered for data interpretation is the interregional variation of HEV prevalence in Italy, where a higher circulation of HEV in Central Italy, especially in Lazio and Abruzzo, than in other regions was observed [10].

However, in CLE, the frequency of patients from Central Italy was similar in the anti-HEV+ and anti-HEV− patient groups, supporting the association between anti-HEV+ and CLE. In contrast, in SLE, the frequency of patients from Central Italy was higher in anti-HEV+ patients than in anti-HEV-.

Another pathogenetic mechanism that needs to be considered is that apoptosis is closely related to the lupus condition. The increased apoptosis and defective clearance of apoptotic cells lead to heightened autoantigen exposure, triggering autoimmune and inflammatory responses in SLE [27]. Increased apoptotic cells were also observed in cutaneous lupus erythematosus [28].

The HEV infection has been reported to cause the activation of the mitochondrial apoptotic pathway [29], thus, in our study, HEV infection in lupus patients could potentiate the mechanism of the defect in cell apoptosis, increasing the inflammatory response.

For this reason, patients affected by systemic and cutaneous lupus erythematosus diagnosed with hepatitis E should be closely followed up with regular monitoring in order to avoid the potential onset of liver injury and the worsening of the autoimmune and inflammatory responses characteristic of the lupus condition.

Concerning the risk factors associated with hepatitis infection, no association of the possible risk factors was reported with HEV+, although, of note, the odds of HEV+ relative to contact with animals and to eating raw seafood were strongly higher than the unit in the CLE group.

Certainly, our results need further confirmation, and other studies should be conducted in larger cohorts of CLE populations to better investigate the HEV infection and prevalence in lupus patients.

## 4. Materials and Methods

### 4.1. Patient Populations

Consecutive SLE and CLE patients attending the Lupus Clinic of the Rheumatology Unit, Sapienza University of Rome (Sapienza Lupus Cohort) were enrolled from September 2018 to September 2019. The diagnosis was performed according to the 2019 EULAR/ACR classification criteria [30]. The study was approved by the local Ethical Committee (Prot. PRE-16/18, 15 January 2018—Istituto Superiore di Sanità and amendment Prot. 13058, 24 April 2019—Prot. 16144, 24 May 2019 Istituto Superiore di Sanità), and patients provided written informed consent. At the enrollment visit, each patient underwent a complete physical examination, and clinical and laboratory data were collected in a standardized, computerized and electronically filled form, including demographics and past medical history with the date of diagnosis, co-morbidities, previous and concomitant treatments. Disease manifestations were recorded according to the above-mentioned classification criteria [30].

Antinuclear antibodies (ANAs) were determined by indirect immunofluorescence assay (IIFA) on HEp-2, anti-dsDNA by IIFA on Crithidia luciliae, ENA (anti-Ro/SSA, anti-La/SSB, anti-Sm, anti-RNP), anti-cardiolipin (anti-CL) of IgG or IgM isotype and anti-Beta2glicoprotein I (anti-Beta2GPI) of IgG or IgM isotype by ELISA. Lupus anticoagulant (LA) was assessed according to the International Society on Thrombosis and Hemostasis guidelines. For all subjects, complement C3 and C4 concentrations were determined by nephelometry. Disease activity was assessed by using the SLE Disease Activity Index 2000 (SLEDAI-2K); chronic damage by SLICC Damage Index (SDI) [31,32]. A peripheral blood sample was collected and then centrifuged with serum recovery. The obtained sera were sent for virological screening to the Department of Infectious Diseases of Istituto Superiore di Sanità (ISS), where sera were stored at −80 °C until to analysis.

### 4.2. Serological and Virological Screening HEV

All serum samples were tested for anti-HEV immunoglobulin Ig G antibodies using commercial enzyme-linked immunosorbent assay (ELISA) kits (Wantai, Biologic Pharmacy Enterprise, Beijing, People’s Republic of China) according to the manufacturer’s instructions. The IgG anti-HEV assays use recombinant antigen expressed from the ORF2 region.

### 4.3. Statistical Analysis

The characteristics of the study population according to the two groups (SLE and CLE) were described by frequencies and percentages for categorical variables and by medians and by both interquartile ranges (IQR) and ranges for continuous variables. We used the chi-square-test—or Fisher-test when appropriate—to compare two categorical variables whilst we applied the Mann–Whitney test for continuous variables. Both univariate and multiple logistic regression models were applied considering CLE (vs. SLE) as the event. Meanwhile, HEV+ vs. HEV− were considered as the exposure variable of interest. Multiple logistic models were applied, adding age and sex as covariates. Finally, simple logistic models were used to explore the risk factors (such as work experience with wild or farm animals, hunting, gardening, vegetable gardening, eating raw seafood, eating uncooked wild boar meat, etc.) eventually associated with HEV+, explored separately in the SLE and CLE groups. All statistical analyses were performed in SAS (version 9.4).

## Figures and Tables

**Table 1:** Logistic models for the event of CLE vs. SLE: part (**a**) univariable analysis; part (**b**) multivariable analysis.

Variables	OR *	(95% CI)	*p*-Value
**part (a)** CLE vs. SLE			
HEV+ vs. HEV−	3.82	(1.37–10.65)	0.010
age (51–74) vs. (18–50)	1.93	(0.94–3.96)	0.072
males vs. females	4.14	(1.57–10.89)	0.004
**part (b)** CLE vs. SLE			
HEV+ vs. HEV−	2.88	(0.96–8.68)	0.060
age (51–74) vs. (18–50)	1.98	(0.88–4.45)	0.098
males vs. females	4.84	(1.72–13.63)	0.003

* OR: odds ratio.

**Table 2:** Odds ratios (ORs) for possible risk factors of HEV+ (vs. HEV-) in CLE (part (**a**)) and SLE (part (**b**)) groups.

**(a) Risk Factors in CLE**	**OR**	**95% CI**	***p*-Value**
contact with animals vs. no	9.70 *	(0.10–961.45)	0.332
eating homemade sausages/uncooked meat vs. no	2.07	(0.22–19.35)	0.524
gardening/vegetable gardening vs. no	0.97	(0.24–3.95)	0.963
eating raw seafood vs. no	8.25 *	(0.38–179.19)	0.179
**(b) Risk Factors in SLE**	**OR**	**95% CI**	***p*-Value**
contact with animals vs. no	2.23 *	(0.02–30.98)	0.679
eating homemade sausages/uncooked meat vs. no	0.45	(0.09–2.29)	0.425
gardening/vegetable gardening vs. no	2.21	(0.46–10.51)	0.319
eating raw seafood vs. no	0.45	(0.09–2.15)	0.319

* Note: Firth’s penalized likelihood regression applied; for all other estimates, maximum likelihood logistic regression was applied.

## Data Availability

Data are reported as Tables and Figure 1 in the text.

## Supplementary Materials

The following supporting information can be downloaded at https://www.mdpi.com/article/10.3390/ijms252011162/s1.
