# Supplementary material for: Hepatitis E Virus Infection in Patients with Systemic and Cutaneous Lupus Erythematosus
Source: Int J Mol Sci. 2024 Oct 17;25(20):11162. doi: 10.3390/ijms252011162 (PMC11509021; doi:10.3390/ijms252011162)
Supplement: Supplementary file 1 [file ijms-25-11162-s001.zip › ijms-3204812-supplementary.pdf]

Table S1. *Demographic, clinical, laboratory and treatment data for the SLE and CLE patients*

	SLE N= 92	CLE N= 46	p-value
**Sex, n (%)**			
Female	84 (91.3%)	33 (71.7%)	**0.003**
Male	8 (8.7%)	13 (28.3%)	
**Age (years)**			
Median (IQR) [range]	47 (39-56) [18-72]	52 (42-62) [31-74]	**0.038**
**Nationality, n (%)**			
Italian	81 (88.0%)	46 (100%)	**0.016**
Foreign	11 (12.0%)	0	
**Education*, n (%)**			
Graduated	25 (29.1%)	9 (19.6%)	0.234
Not gradated	61 (70.9%)	37 (80.4%)	
Unknown	6 (6.5%)**	0	
**Work activity*, n (%)**			
Intellectual	30 (37.0%)	15 (32.6%)	0.616
Not Intellectual	51 (63.0%)	31 (67.4%)	
Unknown	11 (12.0%)**	0	
**Disease duration (years)**			
Median years (IQR) [range]	11 (6- 22) [0 - 40]	10 (4-18) [0.5 - 38]	0.185
**Clinical manifestations #, n (%)**			
Joint involvment	67 (72.8%)	0	
Skin involvment	61 (66.3%)	46 (100%)	**< 0.001**

**Gilliam classification of CLE** §			
acute cutaneous	0	33 (71.7%)	
subacute cutaneous	0	9 (19.6%)	
Lupus profundus	0	1 (2.2%)	
Lupus tumidus	0	3 (6.5%)	
Neuropsychiatric involvement	19 (20.6%)	0	
Hematological manifestations	49 (53.3%)	0	
Sierositis	18 (19.6%)	0	
Renal involvement	25 (27.2%)	0	
**Laboratory manifestations #, n (%)**			
Antinuclear antibodies (ANA)	92 (100%)	25 (54.3%)	**< 0.001**
Anti-dsDNA	57 (62.0%)	0	
Anti-Sm	17 (18.5%)	0	
Anti-SSA	44 (47.8%)	9 (19.6%)	**0.001**
Anti-SSB	20 (21.7%)	0	
Anti-RNP	16 (17.4%)	0	
Anti-cardiolipin IgG/IgM	26 (28.3%)	2 (4.3%)	**0.001**
Anti-β2Glicoprotein I IgG/IgM	18 (19.6%)	0	
Lupus anticoagulant	11 (12.0%)	1 (2.2%)	**0.061**
Low C3 level < 70 (mg/dl)	11 (12.0%)	1 (2.2%)	**0.061**
Low C4 level < 10 (mg/dl)	8 (8.7%)	0	
**Treatments #, n (%)**			
Corticosteroids	82 (89.1%)	11 (23.9%)	**<0.001**
Hydroxychloroquine	88 (95.6%)	42 (91.3%)	0.44
Cyclosporine A	20 (21.7%)	0	
Methotrexate	17 (18.5%)	0	
Cyclophosphamide	12 (13.0%)	0	
Mycophenolate mofetil	19 (22.8%)	0	

Azathioprine	26 (28.3%)	0
Rituximab	7 (7.6%)	0

presence of clinical, laboratory manifestations and used treatments. Therapies are related to disease history

*percentages and p-value unknown excluded ; ** percentages of unknown on the total (n=92)

§ Gilliam classification has been used to define the CLE subsets
